# Santonate of the Protoxide of Mercury—An Excellent Vermifuge

**Published:** 1860-05

**Authors:** 


*Santonate of the Protoxide of Mercury—an excellent Vermifuge.*—When equal parts of nitrate of protoxide of mercury and of santonate of soda,* dissolved separately in a sufficient quantity of distilled water, are mixed together, a large precipitation of proto-santonate of mercury takes place. This is allowed to stand for twenty-four hours, then collected on a filter and washed with distilled water until the latter passes off without taste, whereupon the salt is slowly dried and put in a well-stoppered flask, in which it is kept from the influence of light. It forms a whitish powder, with traces of crystalization, when examined with the aid of a glass; it is odorless, but has a remotely metalic taste, which changes afterwards to bitter and is lasting. It is insoluble in water or alcohol, and is not decomposed at 212° F.; at a high temperature the santonin (santonic acid) is charred and destroyed, while the mecury sublimes partly in the metallic state, partly as peroxide. Exposed to a low heat on filtering-paper it does not fuse nor cause a stain, but become a brownish powder. By lime-water it is decomposed into soluble santonate of lime and black oxide of mercury.†—*Archiv der Pharmacie*, *Nov.* 1859.

**Santonate of Soda* is prepared by digesting an alcoholic solution of santonin with carbonate of soda, evaporating the solution to dryness at a temperature not exceeding 90° F., and redissolving in absolute alcohol, from which solution the salt is obtained by spontaneous evaporation in the form of fine needles.—*Ed. Drug. Circular.*

†The above is an abstract of a translation from the Italian in one of the latest numbers of the *Archiv der Pharmacie* (Organ of the North-German Apothecaries' Union), the superscription being the same as in our translation. No doses, however, are given, and we must suppose that it is to be given in the proportions in which santonin and calomel are used at present. That it possesses superior qualities as a vermifuge may be presumed from its being styled "excellent" by an authority like the *Archiv.—Ed. Drug. Circular.*

